# T cell specific *Cxcr5* deficiency prevents rheumatoid arthritis

**DOI:** 10.1038/s41598-017-08935-6

**Published:** 2017-08-21

**Authors:** Georgios L. Moschovakis, Anja Bubke, Michaela Friedrichsen, Christine S. Falk, Regina Feederle, Reinhold Förster

**Affiliations:** 1 Institute of Immunology, Hannover Medical School, Carl-Neuberg Str. 1, 30625 Hannover, Germany; 2 Institute of Transplant Immunology, Integrated Research and Treatment Center Transplantation, IFB.Tx, Hannover Medical School, Carl-Neuberg Str. 1, 30625 Hannover, Germany; 3 Institute for Diabetes and Obesity, Monoclonal Antibody Core Facility and Research Group, Helmholtz Center Munich, German Research Center for Environmental Health (GmbH), Marchioninistr. 25, 81377 Munich, Germany

## Abstract

The chemokine receptor CXCR5 is primarily expressed on B cells and Tfh cells and facilitates their migration towards B cell follicles. In the present study we investigated the role of the CXCL13/CXCR5 axis in the pathogenesis of rheumatoid arthritis (RA) and specifically addressed the impact of CXCR5-mediated T and B cell migration in this disease. Employing collagen-induced arthritis (CIA) we identify CXCR5 as an absolutely essential factor for the induction of inflammatory autoimmune arthritis. *Cxcr5*-deficient mice and mice selectively lacking *Cxcr5* on T cells were completely resistant to CIA, showed impaired germinal center responses and failed to mount an IgG1 antibody response to collagen II. Selective ablation of CXCR5 expression in B cells also led to suppression of CIA owing to diminished GC responses in secondary lymphoid organs (SLO) and impaired anti-collagen II antibody production. Chimeric mice harboring *Cxcr5*-proficient and *Cxcr5*-deficient immune cells revealed SLO and not the synovial tissue as the compartment where CXCR5-mediated cell migration induces autoimmune inflammation in arthritis. Thus our data demonstrate that CXCR5-mediated co-localization of Tfh cells and B cells in SLOs is absolutely essential for the induction of RA and identify CXCR5 and Tfh cells as promising therapeutic targets for the treatment of RA.

## Introduction

Rheumatoid arthritis (RA) is a chronic inflammatory autoimmune disease mainly affecting peripheral diarthrodial joints with a prevalence ranging from 0.4 to 1.3%^1^. The hallmark of RA is synovial inflammation and hyperplasia that can lead to progressive cartilage and bone erosion, resulting in severe pain and disability. The immunopathogenesis of RA is still not completely elucidated, however it is well established that both innate and adaptive immune system components are crucially involved in RA development and chronicity^2^. A key factor in RA pathophysiology is the production of autoantibodies^3^. Autoantibodies in RA lead to immune complex formation in the joints, complement activation and subsequent recruitment and activation of inflammatory leukocytes^2^. Several autoantibody systems have been identified in RA. Rheumatoid factor (RF) and anti-citrullinated protein/peptide antibodies (ACPAs) can be detected in approximately 50–80% of RA patients and can appear many years prior to the development of arthritic symptoms^4–7^. The presence of RF or ACPAs in serum of RA patients correlates with a higher disease severity and manifestation of systemic extra articular symptoms^8, 9^.

Robust high affinity autoantibody production is usually preceded by activation, somatic diversification and affinity maturation of autoreactive B cells. These processes take place in germinal centers (GC) where activated B cells interact with follicular dendritic cells (FDCs) and T follicular helper cells (Tfh) and upon successful selection differentiate into either memory B cells or antibody secreting plasmablasts^10^. The localization of both B cells and Tfh cells in lymphoid organs is orchestrated by signaling mediated through chemokine receptors such as CCR7, CXCR4, and CXCR5^11^. Expression of CXCR5 allows cells to follow gradients of the chemokine CXCL13 to reach follicular areas. CXCL13 is constitutively produced in B cell follicles and ectopic lymphoid tissue by FDCs and macrophages^12, 13^. Studies on *Cxcr5* ^−/−^ and *Cxcl13* ^−/−^ mice have revealed essential roles of this chemokine axis in lymphoid organ organogenesis and immune cell migration^14–16^. The splenic architecture is altered in *Cxcr5* ^−/−^ mice. B cells are not organized into follicles but form a rim around the T cell zone which is located in the center of the follicle but not polarized as in WT mice^15^. Adoptive transfers of B cells from *Cxcr5* ^−/−^ mice demonstrated a requirement for CXCR5 expression on B cells for successful migration into splenic follicular areas but not for migration into LN follicles^15^. Whereas GCs supporting B cell affinity maturation can be induced in *Cxcr5* ^−/−^ mice they are aberrantly localized in the splenic PALS^15, 17^. These findings suggest that the CXCL13/CXCR5 axis could also be involved in autoimmunity and especially in diseases with ample autoantibody production such as RA. Indeed, CXCL13 levels are increased in sera, synovial fluid and synovial tissue of RA patients and have been proposed to be a reliable marker of synovial inflammation^18–20^. CXCL13 neutralization by anti-CXCL13 mAbs was shown to ameliorate the severity of collagen-induced arthritis (CIA) in mice but anti-collagen II (CII) Ab levels and GC response remained unaffected^21, 22^. Additionally, recent studies show an increased frequency of circulating CXCR5^+^ T cells in peripheral blood of RA patients that correlate with serum ACPA levels and disease severity^23, 24^.

In the present study we investigated the role of CXCR5-mediated cell migration in the induction of RA. By using a model of collagen-induced arthritis (CIA) enabling high disease incidence in the relatively resistant C57BL/6 genetic background we demonstrate that *Cxcr5* ^−/−^ mice were overall resistant to CIA induction, showed no signs of synovial inflammation and exhibited profoundly decreased levels of anti-collagen II antibodies. We show that CXCR5-mediated cell migration was redundant for the formation of the inflammatory infiltrate in arthritic paws but indispensable for robust autoimmune GC responses in secondary lymphoid organs (SLOs) during CIA. Mice with a selective *Cxcr5* deficiency in B cells (B-CXCR5^−/−^) exhibited a highly defective GC- and anti-CII antibody response and profoundly ameliorated CIA incidence and severity. Mice with a selective *Cxcr5* deficiency in T cells (T-CXCR5^−/−^) were characterized by hampered GC formation, very weak antibody response to CII upon CIA induction and decreased serum levels of several pro-inflammatory cytokines. Most importantly, T-CXCR5^−/−^ mice did not develop arthritic paws throughout the observation period. Thus our data suggest that the CXCR5-mediated migration of Tfh cells in B-cell follicles is essential for the induction of RA and that CXCR5 and Tfh cells represent promising therapeutic targets in RA.

## Results

### *Cxcr5*-deficient mice are resistant to the development of CIA

**Figure 1:**
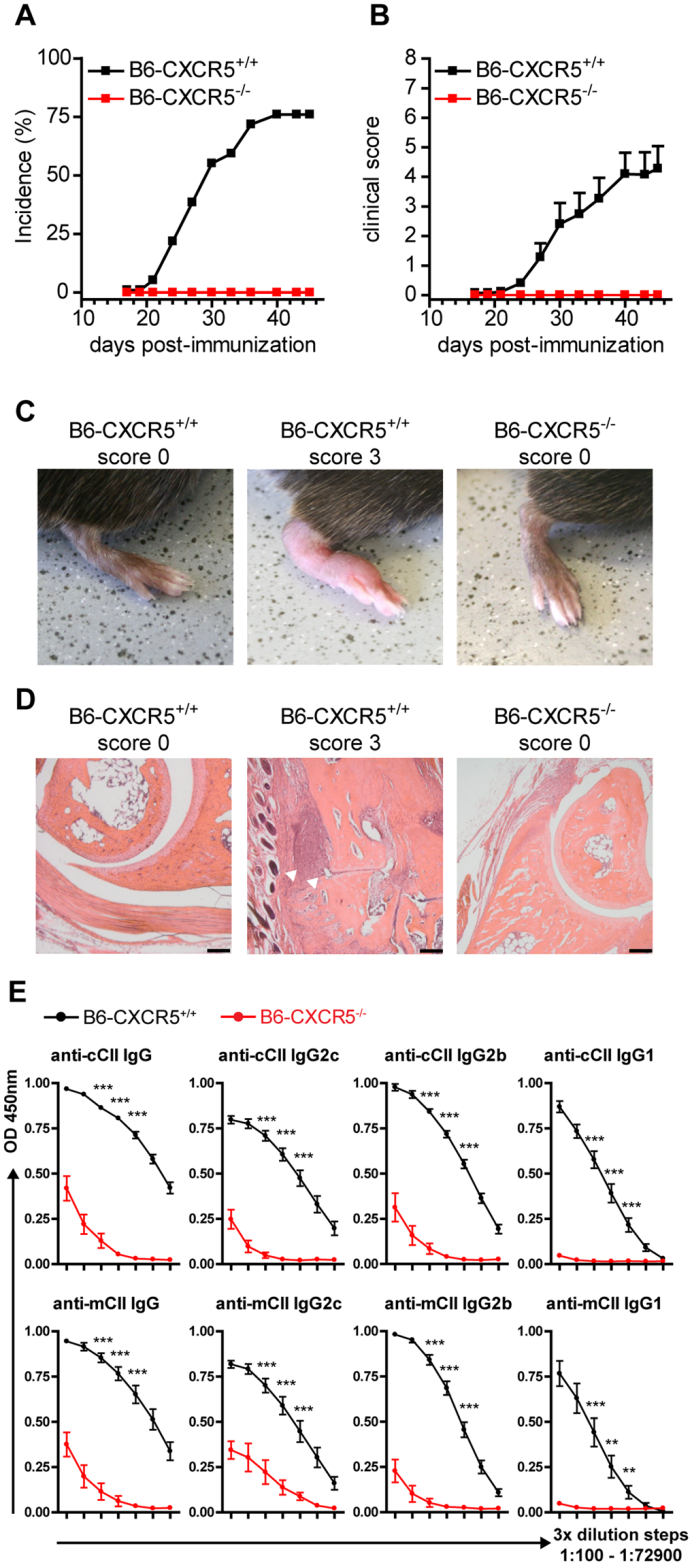
*Cxcr5* ^−/−^ mice are resistant to CIA induction. Incidence (**A**) and clinical score (**B**) of collagen-induced arthritis in WT and *Cxcr5* ^−/−^ mice on the B6 background. Results are shown from ≥12 mice per group. Data in (**B**) are presented as mean ± SEM. Representative images of clinical signs of arthritis (**C**) and histopathology of hind paws (**D**) are shown for healthy and arthritic WT mice and *Cxcr5* ^−/−^ mice. White arrowheads point to areas with inflammatory infiltration. (**E**) Sera were collected at day 45 post-CIA induction from WT and *Cxcr5* ^−/−^ mice and total IgG, IgG1 and IgG2c antibody levels of anti-chicken CII (cCII) Abs (top row) or anti-murine CII (mCII) Abs (bottom row) were measured by ELISA in serially diluted serum samples (3-fold dilution steps, 1:100–1:72900). Results are shown from 8–9 mice per group. Data are presented as mean ± SEM. *P < 0.05; **P < 0.01; ***P < 0.001.

In order to address the role of CXCR5 signaling in the pathogenesis of RA we used a CIA mouse model in *Cxcr5* ^−/−^ mice on a C57BL/6 genetic background. *Cxcr5* ^−/−^ mice were completely resistant to CIA showing no arthritic symptoms throughout the monitoring period (Fig. 1A–C). Histopathologic analysis of joints in paws of *Cxcr5* ^−/−^ mice lacked signs of cellular infiltration, synovial hyperplasia, and joint destruction and joint spaces were well preserved (Fig. 1D). Because the antibody response to CII is an essential pathogenic driver in CIA^25, 26^, serum anti-CII antibody levels were determined. In sera of *Cxcr5* ^−/−^ mice the specific antibody response to both chicken and murine CII was severely suppressed and especially in case of IgG1 antibodies almost absent (Fig. 1E). Taken together these data show that functional CXCR5 signaling is essential for the development of robust anti-CII Ab responses and the development of CIA.

### *Cxcr5* deficiency does not prevent leukocyte migration to arthritic paws

**Figure 2:**
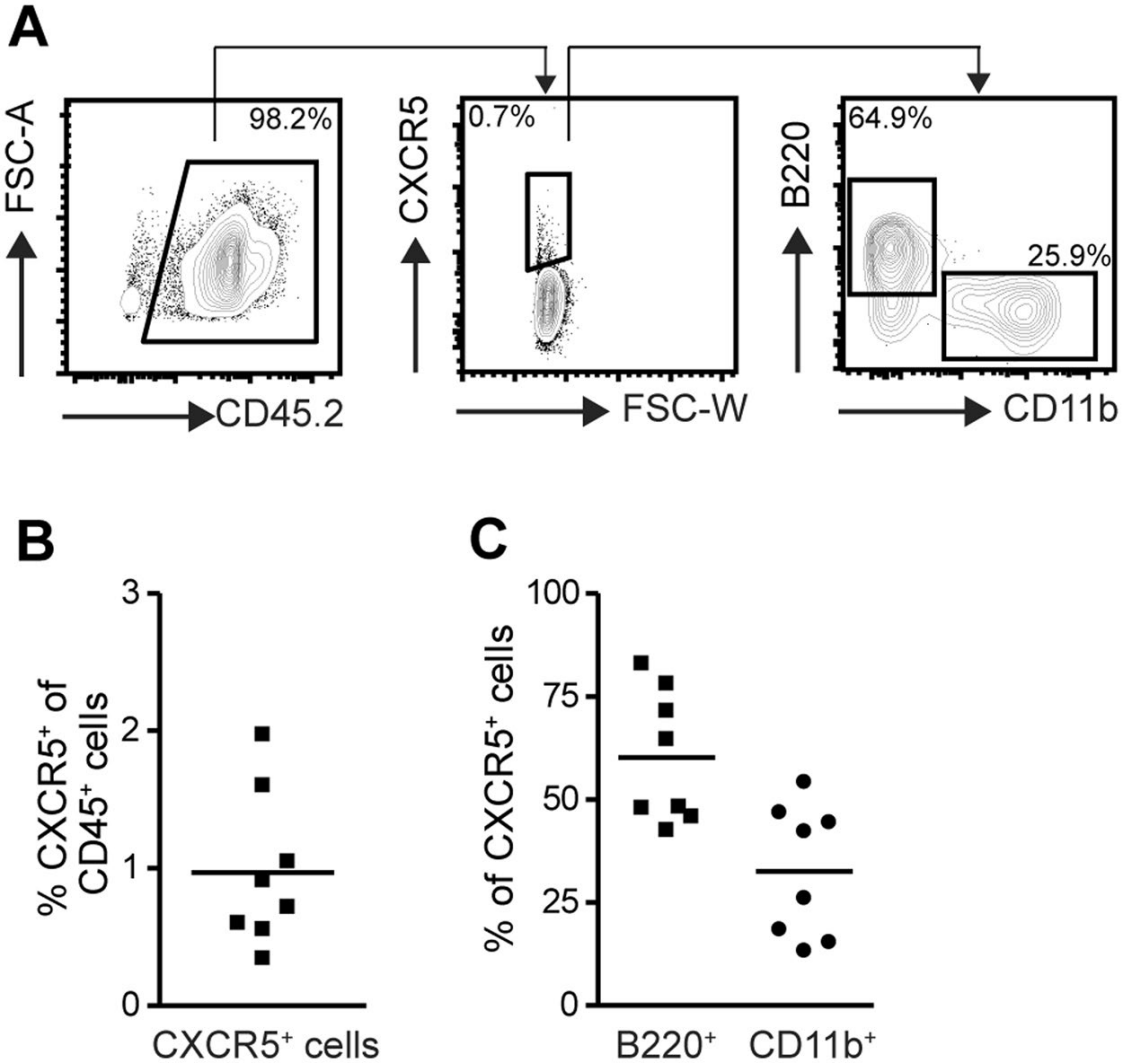
Detection of CXCR5 - expressing cells in murine arthritic paws. (**A**) Representative plots showing identification and characterization of CXCR5^+^ cells in the inflammatory infiltrate of arthritic paws. Cells were pre-gated as DAPI^−^ as shown in suppl. Figure 1. (**B**) Frequency of CXCR5 - expressing cells in arthritic paws of WT mice. (**C**) Frequency of B cells (CD11b^−^ B220^+^) and myeloid cells (CD11b^+^) in the CXCR5^+^ population in arthritic paws. Results are shown from 8 mice.

**Figure 3:**
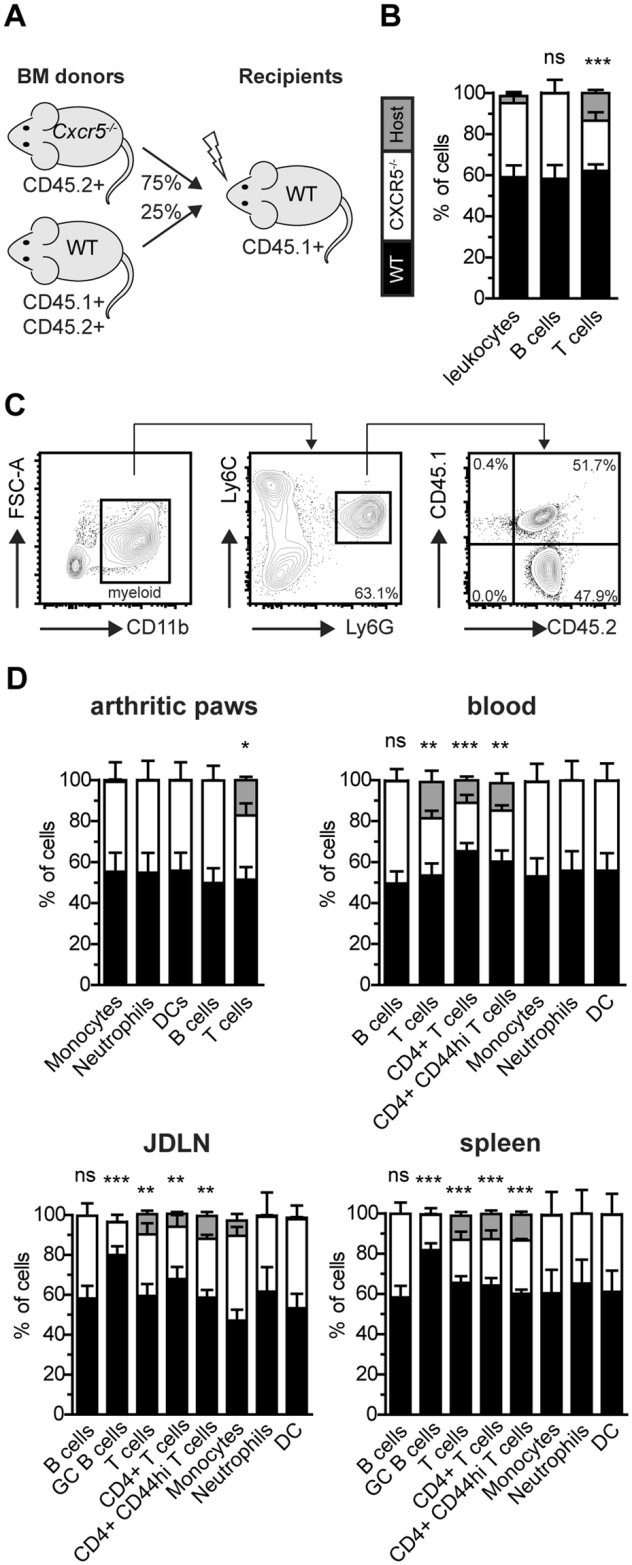
*Cxcr5* deficiency does not influence the composition of the inflammatory infiltrate in arthritic paws. (**A**) Generation of mixed *Cxcr5* ^−/−^/WT chimeric mice. BM from *Cxcr5* ^−/−^ donor mice (CD45.2^+^) was mixed at a ratio of 3:1 with BM from WT donor mice (CD45.1^+^CD45.2^+^) and injected in lethally irradiated WT recipient mice (CD45.1^+^). (**B**) Reconstitution of total leukocytes, B and T cells in peripheral blood of mixed *Cxcr5* ^−/−^/WT chimeric mice before CIA induction. The left bar indicates the color code of cells of given origin. (**C**) Gating strategy for the identification of WT and *Cxcr5* ^−/−^ neutrophils in arthritic paws of mixed *Cxcr5* ^−/−^/WT chimeric mice. The origin of cells, either donor WT, donor *Cxcr5* ^−/−^, or recipient WT, was identified by the expression of CD45.1 and CD45.2. (**D**) Frequencies of donor WT (black bars), donor *Cxcr5* ^−/−^ (white bars) or recipient WT (grey bars) cells for the cell types indicated in arthritic paws, peripheral blood, spleen and JDLN of arthritic mixed *Cxcr5* ^−/−^/WT chimeric mice. Data are presented as mean ± SEM from 7 mice. Frequencies of donor WT and donor *Cxcr5* ^−/−^ cells were compared for statistical significance. Ns, not significant; *P < 0.05; **P < 0.01; ***P < 0.001.

The absence of inflammatory cellular infiltrates in the paws of *Cxcr5* ^−/−^ mice in this CIA model prompted us to investigate the role of CXCR5 in the migration of leukocytes into arthritic joints. The frequency of CXCR5^+^ cells in arthritic paws of WT mice was approximately 1% of all CD45^+^ cells (Fig. 2B). Approximately 65% of all CXCR5 - expressing cells in the inflamed paws were B cells (B220^+^) and some 30% were myeloid cells (CD11b^+^; Fig. 2C). In order to delineate if *Cxcr5* deficiency affects the migration and/or retention of leukocytes in arthritic paws and thereby the composition of the inflammatory infiltrate we generated bone marrow (BM) chimeras reconstituted with a mixture of *Cxcr5* ^−/−^ and WT BM cells (Fig. 3A). Upon development of CIA in the chimeric mice, the cellular composition of the inflammatory infiltrate in affected paws was analyzed by flow cytometry. The origin of cells, either donor WT, donor *Cxcr5* ^−/−^, or recipient, was identified by the use of congenic markers (Fig. 3C). Similar frequencies of cells in the inflammatory infiltrate in arthritic paws were derived from WT or *Cxcr5* ^−/−^ donor mice for monocytes (CD11b^+^ Ly6c^hi^ Ly6G^−^), neutrophils (CD11b^+^ Ly6G^+^), DCs (CD11b^+^ Ly6c^−^ Ly6G^−^ CD11c^+^) and B cells (CD11b^−^ B220^+^; Fig. 3D) but not for T cells, reflecting the diminished reconstitution of *Cxcr5* ^−/−^ T cells in the recipients. The same analysis was performed on cells isolated from peripheral blood, joint-draining lymph nodes (JDLN) and spleen of arthritic WT/*Cxcr5* ^−/−^ mixed chimeras. Neutrophils, monocytes, DCs, and follicular B cells generated from *Cxcr5* ^−/−^ BM were found in these organs at frequencies similar to cells of WT BM origin (Fig. 3D). However in spleen and JDLN the frequency of *Cxcr5* ^−/−^ GC B cells (B220^+^ Fas^+^) was significantly reduced compared to GC B cells of WT origin. Furthermore, significantly lower frequencies of *Cxcr5* ^−/−^ T cells compared to WT T cells were found in JDLN, SPL and peripheral blood (Fig. 3D). These results suggest an essential role for CXCR5 signaling in the generation of the germinal center response in secondary lymphoid organs (SLOs) during CIA but not in the local cellular inflammatory response in arthritic paws.

### Mice with B cell - specific *Cxcr5* deficiency show severely impaired CIA

**Figure 4:**
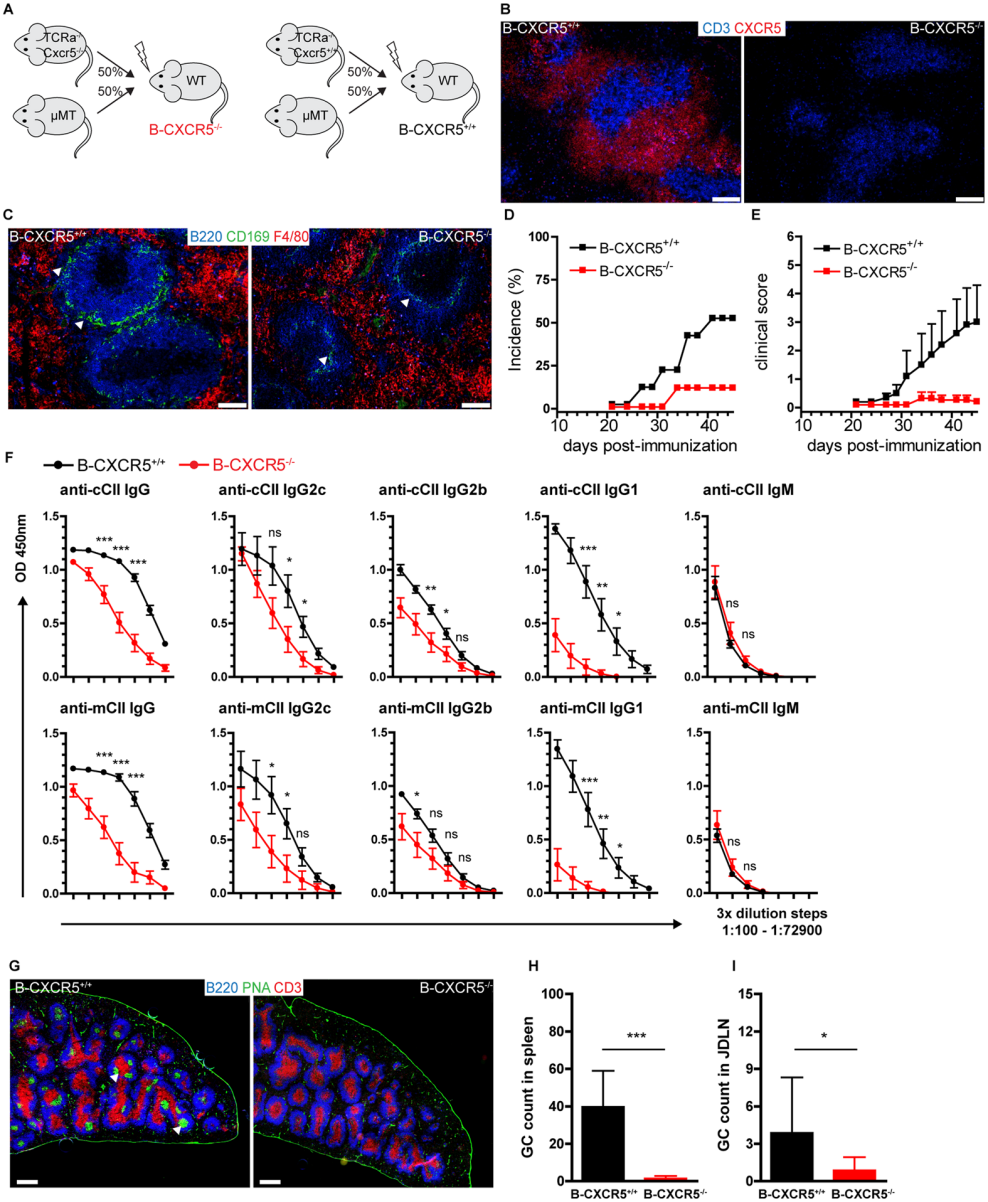
Severely ameliorated CIA in mice with B cell - specific *Cxcr5* deficiency. (**A**) Schematic representation of the generation of B-CXCR5^−/−^ and B-CXCR5^+/+^ mixed chimeric mice. B-CXCR5^−/−^ and B-CXCR5^*+/+*^ mice were generated by reconstitution of lethally irradiated WT recipient mice with BM from *Tcra* ^−/−^ *Cxcr5* ^−/−^ and µMT donor mice and BM from *TCRa* ^−/−^ *Cxcr5* ^*+/+*^ and µMT donor mice respectively. (**B**) Absence of CXCR5 expression on B cells in splenic follicles of B-CXCR5^−/−^ mice. Spleen sections from B-CXCR5^−/−^ and B-CXCR5^+/+^ mice were evaluated for CXCR5- expressing cells using a novel in-house generated anti-murine-CXCR5 mAb (clone 6C3) enabling faithful CXCR5 staining in organ sections (bar 100 µm). (**C**) B cells in spleen of B-CXCR5^−/−^ mice are aberrantly localized outside the marginal zone adjacent to the red pulp. The marginal zone (white arrowheads) was designated by a rim of CD169^+^ macrophages and the red pulp by F4/80^+^ macrophages. Representative pictures from 9–10 mice per group are shown. Bar, 100 µm. Incidence (**D**) and mean clinical score (**E**) of collagen induced arthritis in B-CXCR5^−/−^ and B-CXCR5^+/+^ mixed chimeric mice. Upon a reconstitution period of 10 weeks CIA was induced in B-CXCR5^−/−^ and B-CXCR5^+/+^ mice and mice were monitored for signs of arthritis until day 45 post-immunization. Data are mean ± SEM from 9–10 mice per group. (**F**) Sera were collected from B-CXCR5^−/−^ and B-CXCR5^+/+^ mice upon CIA induction and levels of anti-chicken-CII Abs (top row) and anti-murine-CII Abs (bottom row) were measured by ELISA in serially diluted serum samples (3-fold dilution steps, 1:100–1:72900). Data are shown from 9–10 mice per group. (**G**) Composite micrographs of medial longitudinal splenic sections from B-CXCR5^−/−^ and B-CXCR5^+/+^ mice upon CIA induction (day 45). GCs were identified by PNA staining (white arrowheads). Bar, 500 µm. (**H**) GCs, identified by PNA staining as shown in (G) were counted in composite micrographs of longitudinal medial splenic sections and in sections of JDLN (I) in B-CXCR5^−/−^ and B-CXCR5^+/+^ mice at day 45 upon CIA induction. In (**I**) the number of GCs per analyzed JDLN is shown. Data are presented as mean ± SD, n = 9–10 per group. Ns, not significant; *P < 0.05; **P < 0.01; ***P < 0.001.

CXCR5 is expressed mainly on B cells and Tfh cells^11^. In order to delineate the role of CXCR5 signaling in B cells in CIA we generated mixed BM chimeras with a B cell-specific *Cxcr5* deficiency (B-CXCR5^−/−^; Fig. 4A). In B-CXCR5^−/−^ mice no CXCR5-expressing B cells could be identified in splenic follicles by using a novel anti-murine CXCR5 mAb (clone 6C3) enabling faithful identification of CXCR5 - expressing cells in organ sections by IF microscopy (Fig. 4B). B cells in spleens of B-CXCR5^−/−^ mice failed to form follicles but were aberrantly located outside the marginal sinus (Fig. 4C). B cell-specific *Cxcr5* deficiency severely lowered the incidence and score of CIA. (Fig. 4D,E). Evaluation of the anti-CII antibody response revealed significantly reduced levels of anti-chicken CII as well as anti-murine CII IgG, IgG2c, IgG1 and IgG2b antibodies but unaltered levels of anti-murine CII IgM in sera of B-CXCR5^−/−^ mice (Fig. 4F). Evaluation of the GC response in spleen and JDLN of B-CXCR5^−/−^ mice revealed substantially reduced GC numbers especially in the spleen (Fig. 4G–I). These results suggest an essential role of B cell-expressed CXCR5 in the formation of the GC response and anti-CII antibody production in CIA.

### Mice with T cell - specific *Cxcr5* deficiency are resistant to CIA

**Figure 5:**
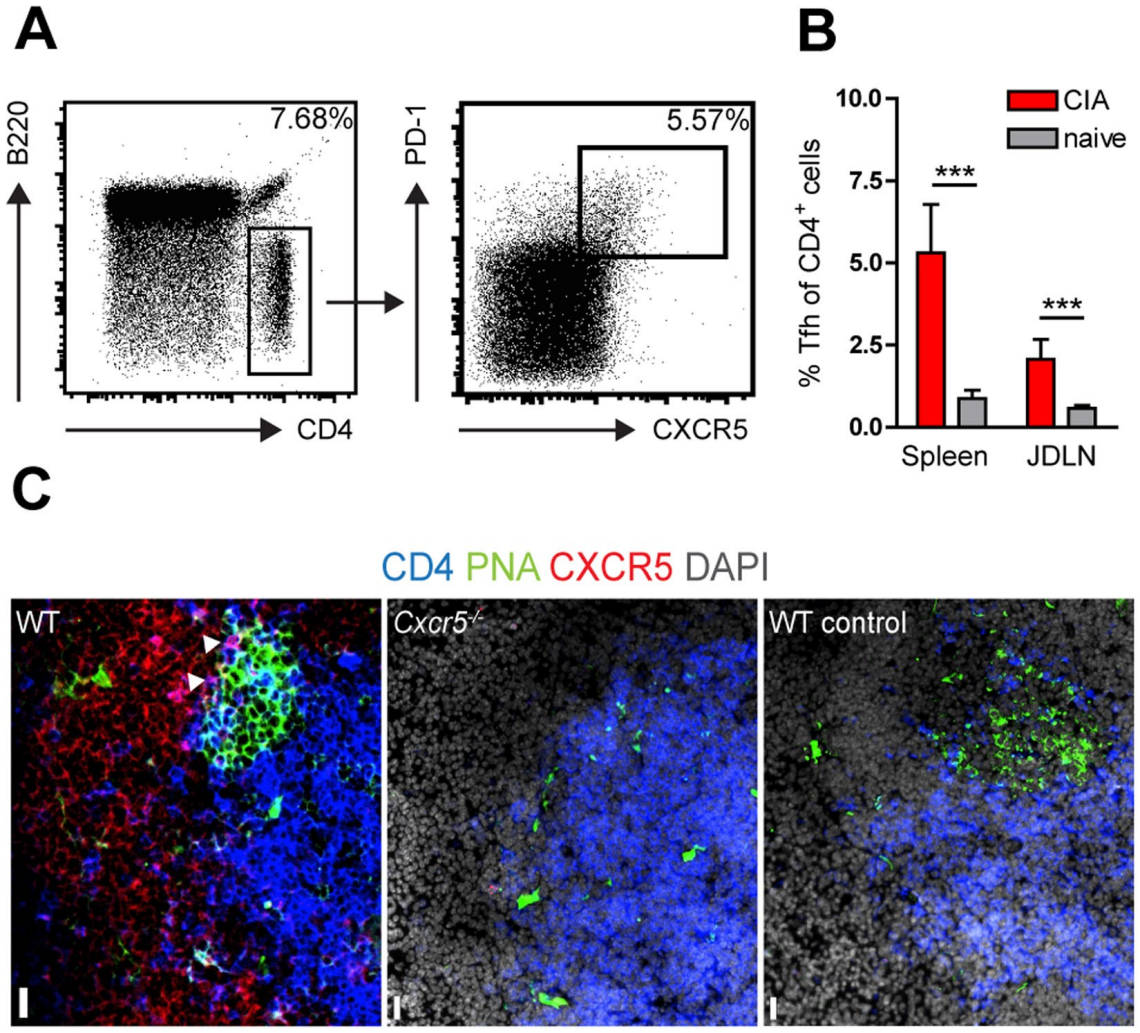
Detection of Tfh cells in SLOs of arthritic BL6 mice. (**A**) Tfh cells were gated as CD4^+^ B220^−^ PD-1^+^ CXCR5^+^ cells. (**B**) Frequency of Tfh cells gated as shown in (**A**) in spleens and JDLN of arthritic BL6 mice (red bars) at day 45 post CII/CFA immunization and non-immunized mice (grey bars). Representative data are presented as mean ± SD (n = 5 mice per group) from more than 2 experiments. ***P < 0.001; (**C**) Tfh cells (CD4^+^ CXCR5^+^, purple color, white arrowheads) were identified by immunofluorescence microscopy in splenic sections of arthritic WT mice (left image) close to GC B cells (PNA^+^) using a novel in-house generated anti-murine-CXCR5 mAb (clone 6C3) (Bar 20 µm). The specificity of the Tfh cell staining was established in splenic sections of *Cxcr5* ^−/−^ mice upon CIA induction (middle image) and in splenic sections of WT arthritic mice with control staining (secondary antibody only, right image). DAPI^+^ CD4^−^ PNA^−^ cells are follicular B cells. Representative data are shown from more than 6 mice analyzed.

**Figure 6:**
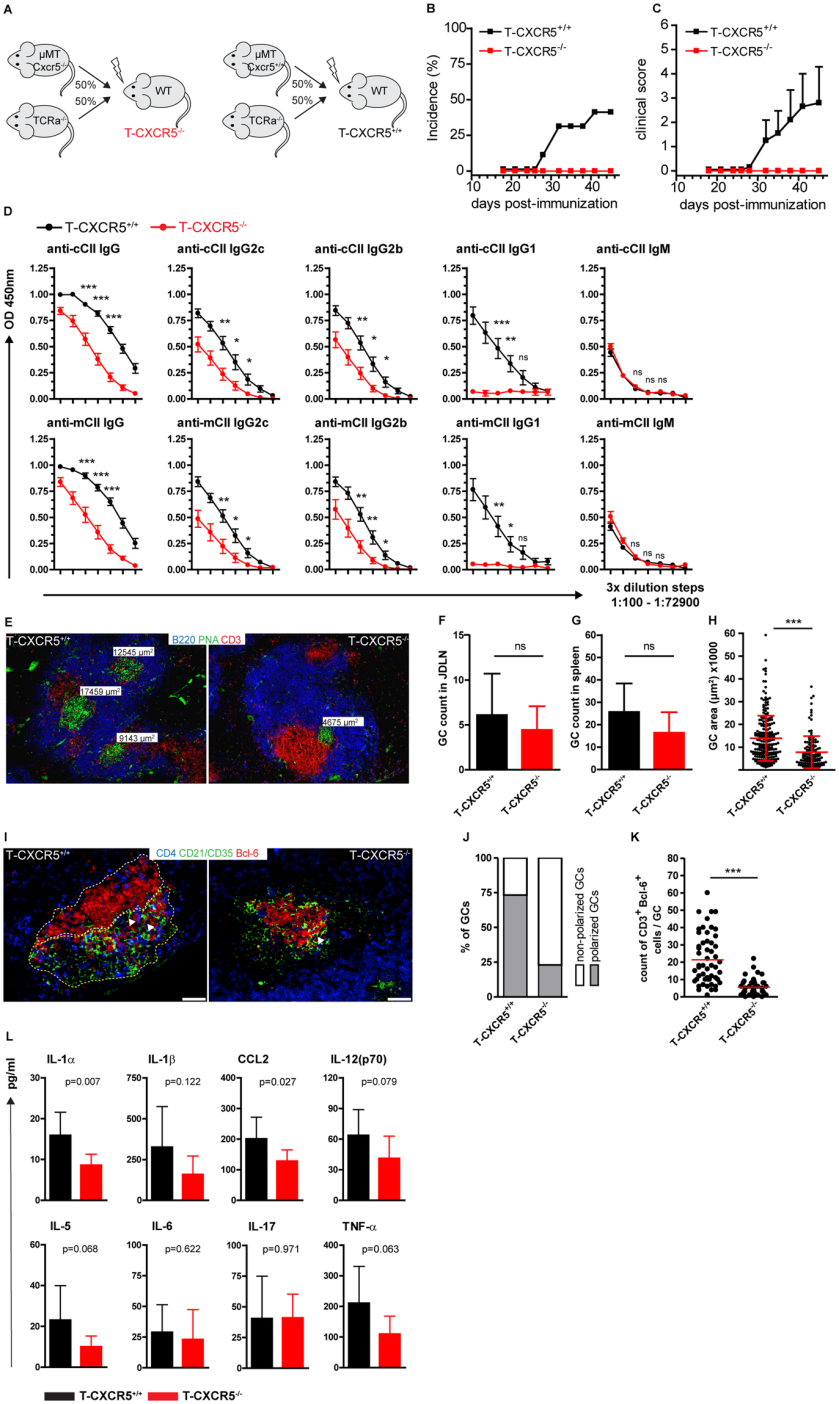
Mice with T cell - specific *Cxcr5* deficiency are resistant to the development of CIA. (**A**) Schematic representation of the generation of T-CXCR5^−/−^ and T-CXCR5^+/+^ mixed chimeric mice. T-CXCR5^−/−^ and T-CXCR5^+/+^ mice were generated by reconstitution of lethally irradiated WT recipient mice with BM from µMT *Cxcr5* ^−/−^ and *Tcra* ^−/−^ donor mice and BM from µMT *Cxcr5* ^*+/+*^ and *Tcra* ^−/−^ donor mice respectively. (**B**) Incidence and (**C**) mean clinical score of CIA in T-CXCR5^−/−^ and T-CXCR5^+/+^ mice. (**D**) Levels of anti-chicken-CII (top row) and anti-murine-CII (bottom row) Abs were measured by ELISA in serial serum dilutions. (**B**–**D**) Data are mean ± SEM from 8–9 mice per group. (**E**) Spleen sections were stained with anti-B220, PNA and anti-CD3 and the area of GCs (PNA^+^) in µm^2^ is indicated. Counts of GCs, identified as shown in (**E**) per JDLN (**F**) and in longitudinal medial sections of spleens (**G**). Data are mean ± SD from 8–9 mice per group. (H) The area of GCs (PNA^+^ regions) was measured in composite micrographs of longitudinal medial splenic sections. Dots represent individual GCs; red bars mean ± SD derived from 8–9 mice per group; ***P < 0.0001; (**I**) JDLN sections were stained with anti-CD4, anti-CD21/CD35 and anti-Bcl-6. The GC dark zone is demarcated by white and the GC light zone by yellow dotted lines. White arrowheads point to Tfh cells (CD4^+^ Bcl-6^+^). Bar 50 µm. (**J**) The frequency of GCs with distinct FDC-rich zone and Bcl-6^+^ GC B cell rich zone (polarized GCs) vs GCs without zone distinction (non-polarized GCs) was assessed in sections shown in (**I**),n ≥ 40 GCs from at least 8 mice per group. (**K**) Sections of JDLN were stained with anti-CD3, PNA and anti-Bcl-6. Number of intra-GC Tfh cells assessed by counting CD3^+^ Bcl-6^+^ cells in Bcl-6^+^ PNA^+^ regions. Data are shown from more than 44 GC analyzed from at least 8 mice per group. ***P < 0.001; Data shown in (**E**–**K**)) were obtained from organs harvested at day 45 post CIA induction; (**L**) Selective reduction of pro-inflammatory cytokines in T-CXCR5^−/−^ mice in CIA. Serum cytokine concentrations were measured in a luminex based assay. Data are presented as mean ± SD from ≥8 mice per group.

Apart from B cells, CXCR5 expression can be identified in mice on CD4^+^ Tfh cells. The frequency of Tfh cells (CD4^+^ CXCR5^+^ PD-1^+^) in CIA was approximately 5.0% in spleen and 2.0% in JDLN (Fig. 5B). Tfh cells were also detected on sections of JDLN of arthritic mice being in close interaction with PNA^+^ GC B cells (Fig. 5C). In order to assess the role of CXCR5 expression on T cells in CIA we used mice with T cell-specific *Cxcr5* deficiency (T-CXCR5^−/−^; Fig. 6A). Interestingly, the incidence of CIA was 0% in T-CXCR5^−/−^ mice throughout the monitoring period (Fig. 6B). Furthermore, the anti-chicken CII as well as the anti-murine CII antibody response were significantly reduced in T-CXCR5^−/−^ compared to T-CXCR5^+/+^ mice for all IgG isotypes analyzed, but not for IgM antibodies (Fig. 6D). Of note, the anti CII IgG1 antibody response was almost absent in T-CXCR5^−/−^ mice. Evaluation of the GC response by immunofluorescence microscopy (Fig. 6E) revealed a slight but not significant reduction in the number of GCs in JDLNs and spleens of T-CXCR5^−/−^ mice (Fig. 6F,G). Assuming that these slight differences in GC numbers might probably not account for the significant reduction of the anti-CII Ab responses observed in T-CXCR5^−/−^ mice in CIA, we measured the size of splenic GCs and evaluated GC morphology in JDLN sections. In spleens of T-CXCR5^+/+^ mice significantly bigger GCs formed compared to spleens of T-CXCR5^−/−^ mice (Fig. 6E and H). In JDLN of T-CXCR5^*+/+*^ mice most GCs exhibited polarization with distinct FDC (CD21/CD35^+^) -rich (light zone) and GC B cell (Bcl-6^+^) -rich (dark zone) areas. In contrast, only 25% of the GCs in JDLN of T-CXCR5^−/−^ mice showed such polarization (Fig. 6I,J). Despite CXCR5 deficiency we were able to detect T cells in GCs of T-CXCR5^−/−^ mice (Fig. 6I) however their numbers were strongly reduced compared to T-CXCR5^*+/+*^ mice (Fig. 6K). The complete resistance to CIA induction in T-CXCR5^−/−^ mice prompted us to investigate the systemic cytokine response. In T-CXCR5^−/−^ mice serum levels of IL-1α and CCL2 were significantly lower compared to T-CXCR5^+/+^ mice (Fig. 6L). Serum levels of IL-1β, TNF-α, IL-5 and IL-12 were non-significantly reduced while other pro-inflammatory cytokines such as IL-6 and IL-17 were not differentially expressed between T-CXCR5^+/+^ and T-CXCR5^−/−^ mice (Fig. 6L). Taken together these data suggest that *CXCR5* expression in T cells is essential for the induction of CIA and has a significant impact on the anti-CII antibody and inflammatory cytokine response during CIA.

## Discussion

The present study reveals a fundamental role for CXCR5 in the pathogenesis of CIA. We provide genetic evidence that expression of this chemokine receptor on B cells as well as Tfh cells plays an essential role for GC formation and function especially during secondary immune responses. So far only scarce mechanistic data has been reported regarding the role of CXCR5 in RA including upregulated CXCR5 expression in human RA synovium^27^. We were able to detect CXCR5-expressing cells in murine arthritic paws in CIA albeit at low frequency. The majority of these cells were B cells and only few CD11b^+^ myeloid cells showed CXCR5 expression. CXCR5 expression on myeloid cells has been previously reported for migratory skin-derived DCs, a subset of marginal zone DCs, DCs in mesenteric LN of *Heligmosomoides* *polygyrus*-infected mice and macrophages in rheumatoid synovium^27–30^. The very few activated T cells we were able to identify by flow cytometry in arthritic paws were CXCR5^−^ (data not shown), similar to most of the activated PD-1^+^ CD4^+^ T cells recently described in RA synovial samples^31^. These CXCR5^−^ ‘peripheral helper’ T cells were shown to localize outside lymphoid aggregates adjacent to B cells and express B cell-help associated factors^31^. Further, we show that leukocytes can migrate to and be retained in the arthritic inflammatory infiltrate independent of CXCR5 and that its expression does not affect the composition of the cellular infiltrate. In contrast, in JDLN and spleens of arthritic mice, CXCR5 expression seems to be essential for the generation of a robust GC B cell response.

Experiments described in this study revealed an outstanding role for the expression of CXCR5 on Tfh cells for the development of CIA. Chimeric mice with a T cell - specific *Cxcr5* deficiency were completely resistant to CIA induction, presented a moderately reduced GC response and showed a severely defective anti-CII antibody production. Furthermore, we could show that during the course of CIA a robust Tfh cell pool is formed in SLOs that probably sustains high anti-CII Ab production. The literature reports conflicting results regarding the role of CXCR5 on T cells in RA models depending on the adoptive transfer of transgenic CD4^+^ T cells carrying a TCR specific for glucose-6-phoshate isomerase (GPI). While one study indicates that CXCR5 expression on T cells is essential for robust disease induction^32^, another group provides evidence for a redundant role for CXCR5 expression on T cells in RA induction and anti-GPI IgG1 production^33^. The reasons for these differences are unclear but it is known that the different genetic backgrounds used in these studies have a major effect on RA initiation and progression. In the present study we used the well characterized CIA model in mice on a clean C57BL/6 genetic background and without the potential bias of out-competition of adoptively transferred CD4 T cells by the endogenous CD4 T cell populations. Using this model we provide strong evidence for an indispensable role of CXCR5 expression on T cells in autoimmune Ab responses and arthritis development. T cell migration into follicular areas was not completely abrogated in this model in the absence of CXCR5 as we were able to detect by IF microscopy occasional *Cxcr5* ^−/−^ T cells in GCs and in extra GC follicular areas (Fig. 6E,I and K). We assume that other chemokine receptors may compensate for *Cxcr5* deficiency and guide T cells to follicular areas or that CCR7 downregulation is sufficient for some *Cxcr5* ^−/−^ activated T cells to enter follicular areas as previously suggested^16, 34^.

An anti-CII IgG1Ab response was virtually absent in *Cxcr5* ^−/−^, *T-CXCR5* ^−/−^ and B-CXCR5^−/−^ mice whereas the anti-CII IgG2 response was reduced but still readily detectable in all these strains. Thus we propose that CIA is highly dependent on an anti-CII IgG1 response, robust GC formation and on Tfh cells, while the IgG2 Ab response is less affected by the absence of CXCR5 and impaired GC formation. Using a murine influenza immunization modeI, it was recently shown that IgG2 responses are mediated by TH1 cells^35^. Interestingly, the TH1 response in T-CXCR5^−/−^ and B-CXCR5^−/−^ mice is most probably unaffected as TH1 cytokines such as IFN-γ and IL-12 were not significantly deregulated in the serum of these mice upon CIA induction in the present study (not shown).

The role of B cells and autoantibodies in RA has gained much attention in recent years in particular due to the therapeutic success of B cell depletion by Rituximab and other drugs^36, 37^. It is widely believed that B cells participate in RA not only by autoantibody production but also through Ag presentation and co-stimulation of autoreactive T cells and through production of pro-inflammatory cytokines^38^. However B cell depletion represents a rather crude approach leading to long term diminution of the overall B cell compartment. Thus there is a clear need for more fine-tuned approaches targeting autoreactive B cells and autoreactive antibody responses. Belonging to the family of seven transmembrane-spanning receptors chemokine receptors have been considered attractive therapeutic targets for interfering with unwanted immune cell trafficking in a variety of immunological disorders including autoimmune diseases. However, most trials targeting chemokine receptors reported largely disappointing results so far^39–41^. Whereas some effects of chemokine receptor inhibitors have been found in tumor settings, HIV infection and animal studies using models of autoimmunity, no promising clinical data on the efficacy of chemokine receptor inhibitors in RA have been reported^41, 42^ but no studies have been described so far targeting CXCR5. This is striking since several studies implicate an important role for CXCR5^+^ cells and the CXCR5/CXCL13 axis in the pathogenesis and progress of several autoimmune conditions including RA. CXCR5^+^ T cells were detected in inflammatory infiltrates in spinal cords of mice with experimental autoimmune encephalomyelitis (EAE) and EAE was attenuated in CXCL13^−/−^ mice^43^. CXCL13 was detected within the perivascular infiltrates in actively demyelinating lesions and was elevated in the cerebrospinal fluid (CSF) of MS patients^44^. Meningeal B cell follicles were associated with severe pathology and disease progress in secondary progressive MS patients and all CSF B cells in samples from MS patients and approximately 20% of CSF CD4^+^ T cells expressed CXCR5^44, 45^. CXCR5 and CXCL13 expression were also readily detected in gut associated lymphoid tissue (GALT) and lymphoid aggregates in gut samples of ulcerative colitis patients. CXCR5^+^ CD4^+^ T cells producing IL-21, a cytokine that promotes mucosal inflammation and B cell activation, were demonstrated in colon samples from IBD patients^46, 47^. In salivary glands and blood of primary Sjögren syndrome (pSS) patients CD4^+^ CXCR5^+^ T cells were shown to be significantly increased^48, 49^. Expansion of circulating CXCR5^+^ Tfh cells (cTfh) with a B cell helper phenotype in the blood has been described besides pSS patients in a variety of autoimmune conditions such as MS, autoimmune thyroiditis, SLE and especially RA^50–55^. The expansion of cTfh cells was associated in most cases with high autoantibody titers and correlated with high disease activity. Especially in RA patients the frequency of cTfh cells showed a positive correlation with anti-CCP Ab levels^23^ and 28-joint count disease activity score (DAS28)^56^.

These studies together with results of the present work suggest that targeting CXCR5 would be an ideal candidate for the treatment of RA for several reasons: i) genetic deficiency for *Cxcr5* completely prevents CIA, a well-established model of RA sharing several immunological patho-mechanisms and genetic susceptibility features with human RA. The CIA model would be well suited in order to verify the efficacy of CXCR5 targeting, being dependent both on T cells and B cells, mainly driven by anti-CII autoantibodies and due to its widespread use to develop current RA therapeutics such as TNFα inhibitors in pre-clinical trials^57^. ii) as CXCL13 is the sole ligand for CXCR5, receptor antagonists have to be only optimized for competing one natural ligand. iii) since only few cell types, primarily B cells and Tfh cells, express CXCR5, targeting of this chemokine receptor would leave large parts of the cellular components of the innate and adaptive immune system unaffected. We propose that targeting CXCR5 in RA would disrupt co-localization of autoreactive B cells and Tfh cells, inhibiting thereby autoreactive GC responses and resulting autoimmune Ab production making the development of CXCR5 inhibitors a promising future approach in RA therapy and probably the therapy of other autoantibody driven autoimmune conditions.

## Materials and methods

### Mice

Mice used were bred at the Central Animal Facility of Hannover Medical School. *Cxcr5* ^−/−^ mice (B6.129S2(Cg)-*Cxcr5* ^*tm1Lipp*^/J) on a BL6 genetic background, B cell deficient µMT (B6.129S2-*Ighm* ^*tm1Cgn*^/J)-, αβ T-cell receptor deficient *Tcra* ^−/−^(B6.129S2-*Tcra* ^*tm1Mom*^/J), B6 CD45.1(B6.SJL-*Ptprc* ^*a*^ *Pepc* ^*b*^/BoyJ)- and B6 CD45.1/CD45.2 heterozygous mice have been described before^16, 58, 59^. µMT *Cxcr5* ^−/−^ and T*cra* ^−/−^ *Cxcr5* ^−/−^ mice were generated by crossing *Cxcr5* ^−/−^ mice to µMT and TCRa^−/−^ mice respectively and were used as bone marrow (BM) cell donors for the generation of mixed BM chimeras as described below. All animals were maintained under SPF conditions. All animal experiments have been performed in accordance with institutional guidelines and approved by the Niedersächsisches Landesamt für Verbraucherschutz und Lebensmittelsicherheit.

### Collagen-induced arthritis and evaluation of arthritis

CIA was induced on a H-2^b^ genetic background as previously described^60^.10- to 14-week-old male mice were used in all experiments. Mice were immunized intradermally at the base of the tail with 100 µg chicken collagen II in dilute acetic acid (MD Bioproducts, Switzerland) mixed with an equal volume of CFA (Difco), 100 µl total injection volume per mouse. 21 days later mice received an identical boost immunization. Mice were monitored and scored several times a week for clinical signs of arthritis as follows: 0 = normal, 1 = mild swelling and erythema affecting single paw joints, 2 = pronounced swelling and erythema affecting one or more paw joints, 3 = severe joint swelling and erythema and ankylosis / joint deformity. The maximum clinical score per mouse was 12.

### Flow cytometry

The isolation of cells from arthritic paws was performed as previously described^61^. Cell suspensions from lymph nodes, spleen, blood and paws were analyzed on a LSR II (BD Biosciences) upon staining with the following mAbs: anti-CD44-eFluor 450 (IM7), anti-CD4 PerCp (RM4-5), anti-PD-1 PE-Cy7 (J43), anti-CD45.2 APC-eFluor 780 (104), anti-CD11b PE-Cy7 (M1/70), anti-Ly-6C PerCp-Cy5.5 (HK1.4), anti-Ly-6G PE (1A8), anti-CD11c PE-Cy7 (N418), anti-CD45.1 APC (A20), anti-CD3e PE-Cy7 (145-2C11), anti-CD62L APC-Cy7 (Mel-14), anti-Fas PE (15A7) (all from eBioscience), anti-CD19 Alexa 488 (6D5) (Biolegend), anti-B220 Pacific Orange (RA3-6B2) (ThermoFisher Scientific), anti-I-Ab FITC (AF6-120.1) (BD Biosciences) and anti-CD11b biotinylated (MAC-1) grown in our laboratories. Staining of biotinylated Abs was followed by incubation with Pacific Orange labeled streptavidin (ThermoFisher Scientific). Expression of CXCR5 was assessed using anti-CXCR5 biotinylated (clone 2G8, BD Biosciences), 30 min at room temperature, followed by APC-eFluor 780 or Cy5 labeled streptavidin (eBioscience). Fluorescence-minus-one (FMO) or isotype control samples were used to assess background fluorescence and unspecific Ab binding. Data were analyzed with FACSDiva (BD Biosciences) and FlowJo Software (Treestar).

### Histological analysis

Paws were fixed in 10% (vol/vol) neutral buffered formalin, decalcified in EDTA and embedded in paraffin blocks. Paw sections (5 µm) were stained with H&E for microscopic evaluation. The degree of inflammatory infiltration, synovial hyperplasia and cartilage/bone erosion was assessed in a blinded fashion. For immunohistological analysis of SLOs, spleen and joint draining lymph nodes were embedded in OCT and frozen on dry ice. 8 µm thick sections were prepared on a cryostat (CM3050; Leica) and fixed for 10 min in ice-cold acetone. Cryosections were rehydrated in Tris-buffered saline with 0.05% Tween 20, blocked with 5% mouse or rat serum and stained with the following antibodies at room temperature: anti-CD3 Cy3 (17A2), anti-CD3 Cy5 (17A2), anti-CD4 Cy5 (GK1.5), anti-CXCR5 (6C3), anti-B220 Cy5 (RA3-3A1) (all grown and labeled in our laboratories), anti-F4/80 PE (BM8) (ThermoFisher Scientific), anti-CD169 FITC (MOMA-1) (AbD Serotec), anti-CD21/CD35 FITC (7G6) (BD Biosciences) and anti-Bcl-6 (7D1) (Santa-Cruz) followed by anti-rat IgG Cy3 (Jackson Immunoresearch). Germinal centers were identified using PNA biotinylated (Sigma-Aldrich) followed by Streptavidin Alexa 488 (ThermoFisher Scientific). Images were acquired using a motorized epifluorescence microscope (BX61; UPlanSApo lenses: 10x/0.4, 20x/0.75 and 40x/0.9) with a fluorescence camera (F-View II) and cellSens software (Olympus). For analysis of the germinal center response longitudinal sections from the middle of the spleen were used, composite images of 10x magnification were produced and the number and area of germinal centers determined.

### Cytokine measurement

Levels of cytokines and chemokines in mouse sera were measured in a Luminex-based cytokine array according to manufacturer’s instructions (Bio-Rad Laboratories, Hercules, USA).

### ELISA

Levels of anti-chicken collagen II and anti-murine collagen II antibodies were assessed in serial dilutions of sera by ELISA. 96-well immunosorbent plates (Nunc, Germany) were coated with 2 µg/ml chicken- or murine- collagen II (MD Bioproducts, Switzerland and Chondrex, Inc. USA, respectively) and anti-collagen II antibodies in mouse sera were detected using HRP conjugated anti-mouse-IgM (ThermoFisher Scientific), biotinylated anti-mouse -IgG (Jackson Immunoresearch), -IgG1, -IgG2b (BD Biosciences)and -IgG2c antibodies (Bethyl Laboratories). Upon incubation with peroxidase-conjugated streptavidin (Jackson Immunoresearch) followed by tetramethylbenzidine (TMB) substrate (Sigma Aldrich) optical density (OD) was measured at 450 nm in an automated microplate reader (Tecan).

### Bone-marrow chimeras

Recipient C57BL/6 male mice were irradiated lethally with a single dose of 9 Gy and reconstituted by i.v. injection of 10 × 10^6^ bone marrow cells isolated from male donor mice. Reconstitution of the lymphoid compartment was assessed 7–8 weeks later by flow cytometric analysis of peripheral blood. 9–10 weeks upon reconstitution collagen-induced arthritis was induced as described above. Mice with a B cell-specific *Cxcr5* deficiency, designated B-CXCR5^−/−^ mice were generated by reconstitution of lethally irradiated male WT recipients with 50% B cell-deficient µMT BM cells and 50% *Tcra* ^−/−^ *Cxcr5* ^−/−^ BM cells. In these recipients T cells were generated from the µMT BM cells that cannot give rise to B cells and *Cxcr5* ^−/−^ B cells from the *TCRα* ^−/−^ *Cxcr5* ^−/−^ BM cells. Other hematopoietic lineages were contributed by both groups of BM cells. Chimeric mice harboring *Cxcr5*-proficient B cells (B-CXCR5^+/+^) were generated similarly by reconstitution with 50% µMT BM cells and 50% *Tcrα* ^−/−^ BM cells. Mice with a T cell specific *Cxcr5* deficiency, designated T-CXCR5^−/−^ mice were generated by reconstitution of lethally irradiated male WT recipient mice with BM cells from B cell-deficient *Cxcr5*-deficient (µMT *Cxcr5* ^−/−^) mice and αβ TCR-deficient (*Tcrα* ^−/−^) mice mixed at a ratio of 1:1. In this setup *Cxcr5* ^−/−^ T cells are derived from µMT *Cxcr5* ^−/−^ BM cells and B cells from the *Tcrα* ^−/−^ BM cells. Other hematopoietic lineages were generated by both groups of BM cells. For the generation of T-CXCR5^+/+^ control mice BM cells from µMT *Cxcr5* ^+/+^ and *Tcrα* ^−/−^ mice were mixed at a 1:1 ratio and injected in lethally irradiated male WT recipient mice.

### Generation of anti-murine-CXCR5 antibodies

Wistar rats were immunised subcutaneously (s.c.) and intraperitoneally (i.p.) with a mixture of membrane fractions isolated from murine *Cxcr5* transfected RBL cells in 500 µl PBS, 5 nmol CpG2006 (TIB MOLBIOL, Berlin, Germany), and 500 µl incomplete Freund’s adjuvant. 6 weeks later, a boost without Freund’s adjuvant was given i.p. and s.c. 3 days before fusion. Fusion of the myeloma cell line P3 × 63-Ag8.653 with the rat immune spleen cells was performed using polyethylene glycol 1500 according to standard procedures^62^. Supernatants were tested by ELISA for IgG production and analyzed by flow cytometry for specificity using lymphocyte suspensions isolated from WT and *Cxcr5* ^−/−^ mice. Hybridoma cells from supernatants that reacted specifically were subcloned at least twice by limiting dilution. Experiments in this study were performed with clone 6C3 (IgG2a/k).

### Statistics

Statistical analysis was performed with Prism 4 (Graph-Pad Software, Inc.). Statistical significance between groups was determined using unpaired two-tailed Student’s *t* test. *P < 0.05; **P < 0.01; ***P < 0.001.

### Data Availability

The datasets generated during and/or analysed during the current study are available from the corresponding author on reasonable request.

## Electronic supplementary material

Supplementary Figure 1
