# Supplementary material for: T cell specific Cxcr5 deficiency prevents rheumatoid arthritis
Source: Sci Rep. 2017 Aug 21;7:8933. doi: 10.1038/s41598-017-08935-6 (PMC5567121; doi:10.1038/s41598-017-08935-6)
Supplement: Supplementary file 1 — Supplementary Figure 1 [file 41598_2017_8935_MOESM1_ESM.pdf]

Supplementary Information

T cell specific *Cxcr5*-deficiency prevents rheumatoid arthritis

Georgios L. Moschovakis, Anja Bubke, Michaela Friedrichsen, Christine S. Falk, Regina Feederle, Reinhold Förster

Supplementary figure 1

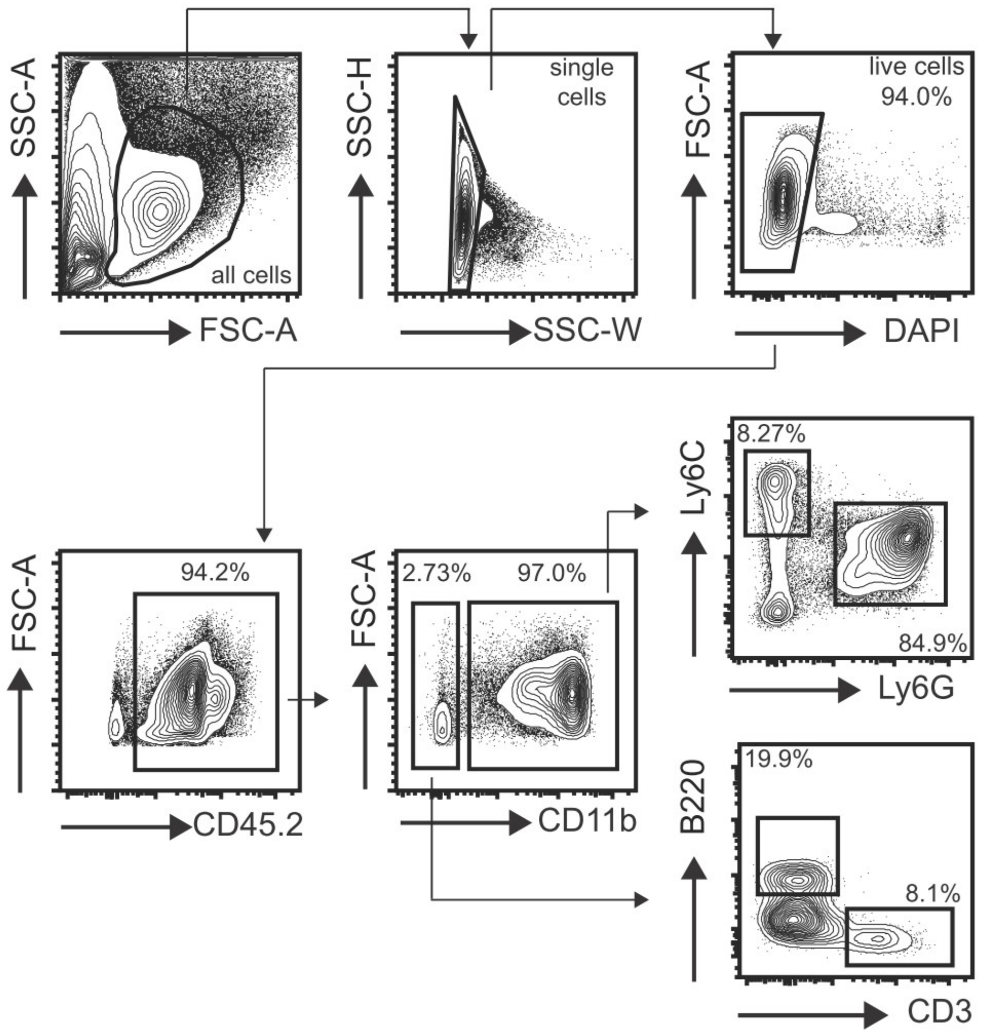

Supplementary Figure 1: Flow cytometric characterization of the inflammatory infiltrate in arthritic hind paws of WT mice. Single cell suspensions from arthritic hind paws were generated as described in material & methods. Representative plots are shown from more than 8 mice analyzed.
